# Complex-tensor theory of simple smectics

**DOI:** 10.1038/s41467-023-36506-z

**Published:** 2023-02-24

**Authors:** Jack Paget, Marco G. Mazza, Andrew J. Archer, Tyler N. Shendruk

**Affiliations:** 1 Interdisciplinary Centre for Mathematical Modelling and Department of Mathematical Sciences, Loughborough University, Loughborough, Leicestershire LE11 3TU UK; 2 Max Planck Institute for Dynamics and Self-Organization (MPIDS), Am Faßberg 17, D-37077 Göttingen, Germany; 3 School of Physics and Astronomy, The University of Edinburgh, Peter Guthrie Tait Road, Edinburgh, EH9 3FD UK

**Keywords:** Liquid crystals, Coarse-grained models, Structure of solids and liquids, Applied mathematics

## Abstract

Matter self-assembling into layers generates unique properties, including structures of stacked surfaces, directed transport, and compact area maximization that can be highly functionalized in biology and technology. Smectics represent the paradigm of such lamellar materials — they are a state between fluids and solids, characterized by both orientational and partial positional ordering in one layering direction, making them notoriously difficult to model, particularly in confining geometries. We propose a complex tensor order parameter to describe the local degree of lamellar ordering, layer displacement and orientation of the layers for simple, lamellar smectics. The theory accounts for both dislocations and disclinations, by regularizing singularities within defect cores and so remaining continuous everywhere. The ability to describe disclinations and dislocation allows this theory to simulate arrested configurations and inclusion-induced local ordering. This tensorial theory for simple smectics considerably simplifies numerics, facilitating studies on the mesoscopic structure of topologically complex systems.

## Abstract (web summary)

As lamellar materials, smectics exhibit both liquid and solid characteristics, making them difficult to model at the mesoscale. Paget et al. propose a complex tensor order parameter that reflects the smectic symmetries, capable of describing complex defects including dislocations and disclinations.

## Introduction

**Fig. 1:**
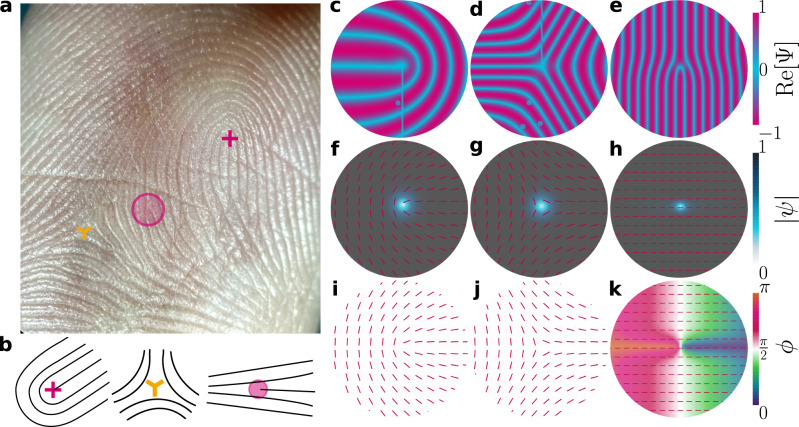
A catalog of defects in 2D smectics. **a** Single defects marked on a high resolution photo of a fingerprint. +1/2 disclination, −1/2 disclination and edge dislocation marked by red cross, yellow trilateral and red circle, respectively. **b** Schematics of (left) +1/2 disclination, (center) −1/2 disclination and (right) dislocations. **c**–**k** Simulations of defects for model parameters *A* = − 1 (lamellar state), *C* = 2 and *κ*^2^ = 0.75 in circular domains with boundary conditions requiring single defects. Columns present three defect types: (first column) + 1/2 disclination; (second) − 1/2 disclination; (third) edge dislocation. Rows show plots of: **c**–**e** layer visualization ${{{{{{{\rm{Re}}}}}}}}\left[{{\Psi }}\right]$; **f**–**h** modulus $\left|\psi \right|$ with **N** overlaid; **i–k** phase *ϕ* with **N**.

Layered materials are key components in many technological, biological and fluidic systems. Graphene, MXene and other two-dimensional materials are composed of atomistically thin sheets with only weak out-of-plane bonding^1^. Moreover, layering is a widespread strategy for increasing the surface area of organs, as in the friction ridges that make up fingerprints (Fig. 1a), convolutions within the cortex of human brains^2^, and the enormous surface area of intestinal villi^3^. At the subcellular level, the Golgi apparatus, rough endoplasmic reticulum, and crista in mitochondria all possess many folds and creases. Layered fluids include liquid crystalline cholesterics which form “pseudolayers” via their helicity^4^ and, most plainly, smectics composed of stacks of orientationally aligned molecules that maintain fluidic disorder within each layer^5^.

The lamellar structure of stacked layers produces fascinating properties. The smectic ground state consists of flat, equally spaced, layers for which both translational and rotational symmetries are broken. Because layers locally break translational symmetry, dislocation-type defects are allowed^6–8^, while broken rotational symmetry of the layering direction results in a singularity of the layer normal, allowing disclination-type defects^6,9^ (Fig. 1b). This makes smectics excellent systems for exploring self-assembly^10–12^ and topology^13–15^, especially in confining geometries^16–18^ or in contact with micropatterned structures^19^.

However, the very properties that make these lamellar phases so interesting also contrive to make them challenging to model. Any order parameter that contends to describe simple smectics, i.e., lamellar phases without underlying nematic order, must explicitly include three pieces of information: (i) the degree of ordering, (ii) dilation/contraction of layers’ spacing and (iii) the direction/bending of layers, either through its instantaneous value or gradients. Since the 1970s, it has thus been recognized that a complex scalar order parameter can be employed to incorporate this information^20^, which leads to a strong analogy to superfluids and superconductors^21–24^. However, such a complex order parameter faces many difficulties^25–27^. Fundamentally, these issues arise because complex scalar order parameters assume a global layer normal direction is known and that the layers only vary about this well-defined direction.

To circumvent this shortcoming, theoreticians have modeled smectic-A liquid crystals by coupling models of smectic ordering to the nematic order parameters for the orientation of rod-like molecules^28–31^. While sensible for smectic liquid crystals with inner-layer nematic ordering, this does not fundamentally solve the issue for many non-nematic lamellar materials, which have their own intrinsic rotational symmetry breaking. Furthermore, these approaches model smectics at the microscopic level, resolving each layer at length scales comparable to the molecular size. This makes such models computationally expensive for mesoscale and hydrodynamic modeling.

Recent studies of defect annihilation in block copolymer films^32,33^ and confined smectic colloidal liquid crystals showing unique pairs of quarter-charge topological defects connected by domain wall bridges^34,35^ motivate the need for alternative theoretical descriptions for simple smectics that allow simulations to tackle more topologically complex structures without relying on microscale models. To create a more general theory for layered materials, we consider “simple” smectics, an idealization of purely lamellar materials that do not simultaneously require a strong degree of orientational alignment of mesogens. We seek a mesoscopic order parameter for such simple smectics that can incorporate both the smectic ordering and layer orientation, and describe our theory as ‘mesoscopic’ because it neglects details of the material structure on molecular (microscopic) length scales, but is able to describe defects as well as the bulk (macroscale) ordering. Here, we propose a formalism to model defect-rich lamellar systems through a complex tensor order parameter that describes the local degree of lamellar ordering, layer displacement and layer normal orientation.

A lamellar system is a periodic variation of the density in a single spontaneously symmetry-broken direction. Traditionally, smectic ordering is approximated by expanding the molecular density at each point **r**, as $\rho \left({{{{{{{\bf{r}}}}}}}},\, t\right)=\mathop{\sum }\nolimits_{m=-\infty }^{\infty }{\psi }_{m}{e}^{im{{{{{{{\bf{q}}}}}}}}\cdot {{{{{{{\bf{r}}}}}}}}}\, \approx \,{\rho }_{0}+2{{{{{{{\rm{Re}}}}}}}}\left[{{\Psi }}\right]$, where *ρ*_0_ is the mean and the microscopic density variation is defined to be the real part of a plane wave with ${{\Psi }}\left({{{{{{{\bf{r}}}}}}}},\, t\right)=\left|\psi \right|{e}^{i\left({{{{{{{\bf{q}}}}}}}}\cdot {{{{{{{\bf{r}}}}}}}}+{\varphi }_{0}\right)}$. Commonly employed in Landau free energy expansions^36,37^, the modulus $\left|\psi \left({{{{{{{\bf{r}}}}}}}},\, t\right)\right|$ quantifies the extent of layering, *φ*_0_ is the phase at the origin, and ${{{{{{{\bf{q}}}}}}}}\left({{{{{{{\bf{r}}}}}}}},\, t\right)$ is the wave vector. In the absence of any deformations, the wave vector **q**_0_ = *q*_0_**N** points in the direction of the layer normal ${{{{{{{\bf{N}}}}}}}}\left({{{{{{{\bf{r}}}}}}}},\, t\right)$ and describes equally spaced layers of thickness 2*π*/*q*_0_. If the layers are locally displaced from their ground state by a layer displacement field ${{{{{{{\bf{u}}}}}}}}\left({{{{{{{\bf{r}}}}}}}},\, t\right)$ that varies slowly on large length and time scales, the argument of the plane wave can be written as ${{\Phi }}\left({{{{{{{\bf{r}}}}}}}},\, t\right)={{{{{{{\bf{q}}}}}}}}\left({{{{{{{\bf{r}}}}}}}},\, t\right)\cdot {{{{{{{\bf{r}}}}}}}}\approx {{{{{{{{\bf{q}}}}}}}}}_{0}\cdot \left({{{{{{{\bf{r}}}}}}}}+{{{{{{{\bf{u}}}}}}}}\right)$. However, the layer displacement must also be parallel to **N** by definition since the simple smectic is isotropic within layers. Thus, the phase $\phi \left({{{{{{{\bf{r}}}}}}}},\, t\right)\equiv {{{{{{{{\bf{q}}}}}}}}}_{0}\cdot {{{{{{{\bf{u}}}}}}}}$ encodes the extent of layer displacement in units of the unperturbed wave number *q*_0_. Variation of $\phi \left({{{{{{{\bf{r}}}}}}}},\, t\right)$ indicates lamellar compression/dilation deformations (hereafter referred to jointly as compression). The symmetries of simple smectics can be described as **N** → − **N**, Φ → Φ + *m*2*π* for $m\in {\mathbb{Z}}$, and Φ → − Φ. This last equivalence relation is identical to Ψ → Ψ^*^. We note that equations such as the Swift–Hohenberg^38^, phase field crystals^39–43^ and density functional theory^44–47^ can model striped phases via a continuous field approach that goes beyond the traditional simplistic sinusoidal approximation. However, these all must resolve individual layers, necessarily limiting their applicability to short times and length scales. Though traditionally the microscopic variation is approximated as the lowest Fourier component of the density, the imperative conclusion is that the (i) degree of layering, (ii) layer displacement, and (iii) layer normal direction are the crucial aspects of the lamellar ordering in the continuum limit that vary on mesoscopic scales, regardless of the microscopic details.

Though elegant and economical, the traditional formalism has known shortcomings^26^. These can be stated in a number of equivalent ways: (i) Only the variation of the density $\sim {{{{{{{\rm{Re}}}}}}}}\left[{{\Psi }}\right]=\left|\psi \right|\cos ({{\Phi }})$ is physical, and so the wave function is not truly a single-valued function of position^23,48^ but rather both Ψ and Ψ^*^ appear simultaneously^27^. (ii) This can be stated as Φ is not an element of the unit circle *S*^1^ but rather of the orbifold ${S}^{1}/{{\mathbb{Z}}}_{2}$^25,49,50^. (iii) The plane wave is a linear function of the layer normal **N**, which does not faithfully reflect the apolar symmetry of the layering. While the phase $\phi \left({{{{{{{\bf{r}}}}}}}},\, t\right)=({q}_{0}{{{{{{{\bf{N}}}}}}}})\cdot (u{{{{{{{\bf{N}}}}}}}})$ is an even function of the layer normal, the first term of ${{\Phi }}\left({{{{{{{\bf{r}}}}}}}},\, t\right)={q}_{0}{{{{{{{\bf{N}}}}}}}}\cdot {{{{{{{\bf{r}}}}}}}}+\phi$ is not. Commonly, the layer normal is defined as a vector via the gradients ${{{{{{{\boldsymbol{\nabla }}}}}}}}{{\Phi }}/\left|{{{{{{{\boldsymbol{\nabla }}}}}}}}{{\Phi }}\right|$, but this is only self-consistent when variations of *ϕ* are negligible. By explicitly considering microscopic density variation on the scale of individual layers^26,31,41^, these issues can be avoided with computational cost, but a hydrodynamic-scale theory that both circumvents these issues and considers only mesoscopic variations of the lamellar order has not previously been established.

In contrast, hydrodynamic-scale nematic theory uses the tensor ${{{{{{{\bf{Q}}}}}}}}=S\left({{{{{{{\bf{n}}}}}}}}\otimes {{{{{{{\bf{n}}}}}}}}-{{{{{{{\boldsymbol{\delta }}}}}}}}/d\right)$ to collect both the scalar order parameter *S* and apolar director **n** into a single order parameter, for dimensionality *d* and identity matrix **δ**^51^. The nematic order parameter simultaneously describes the extent of phase ordering and local direction of broken symmetry in an arbitrary reference frame. Thus, both the bulk and deformation free energy densities can written in terms of ${{{{{{{\bf{Q}}}}}}}}\left({{{{{{{\bf{r}}}}}}}},\, t\right)$. Practically, the introduction of **Q** has enabled numerical simulations of confined nematics^52,53^, colloidal liquid crystals^54–56^ and active fluids^57–59^, by treating defects as locally disordered cores, rather than singularities. Explicitly, the tensor **Q** regularizes these singularities in **n** and remains continuous at the center of defects. In this manuscript, we show how a similar tensorial order parameter can be constructed for a hydrodynamic-scale description of simple smectics that naturally respects the apolar symmetry of layering, the extent of layering and the relative layer displacement.

## Results

In this article, we propose a tensorial order parameter field for simple, lamellar smectics. The tensor ${{{{{{{\bf{E}}}}}}}}\left({{{{{{{\bf{r}}}}}}}},\, t\right)$ is complex, symmetric, traceless and globally gauge invariant. It incorporates the extent of layering and relative layer displacement, as well as the layer normal orientation. It encompasses the advantages **Q**-tensor formalism provides to nematics but for simple smectics. Here, we exclusively consider the simplest smectics, with only lamellar broken translational symmetry and layer-normal broken rotational symmetry. Such simple lamellar ordering is observed in systems with little-to-no nematic ordering, such as the striped phases of short-range attraction and long-range repulsion colloidal systems^44,60,61^, ionic liquid crystals (which commonly transition directly from isotropic to smectic phases)^62^ and the flat plate ("lasagna”) phase of nuclear pasta^63^. We focus solely on this idealistic premise for lamellae to demonstrate the fundamental suitability of **E** for avoiding the phase ambiguity and to show its utility in simulating confining geometries.

We consider only the mesoscopic aspects of simple smectics by rearranging the wave function as ${{\Psi }}=\left|\psi \right|{e}^{i\phi }{e}^{i{{{{{{{{\bf{q}}}}}}}}}_{0}\cdot {{{{{{{\bf{r}}}}}}}}}$. In this form, the wave function possesses two distinct factors: (i) The local microscopic ground state wave ${e}^{i{{{{{{{{\bf{q}}}}}}}}}_{0}\cdot {{{{{{{\bf{r}}}}}}}}}$ and (ii) the mesoscopic complex order parameter $\psi \left({{{{{{{\bf{r}}}}}}}},\, t\right)=\left|\psi \right|{e}^{i\phi }$. Both the extent of layering $\left|\psi \right|\left({{{{{{{\bf{r}}}}}}}},\, t\right)={\left(\psi {\psi }^{*}\right)}^{1/2}$ and the phase $\phi \left({{{{{{{\bf{r}}}}}}}},\, t\right)$ are assumed to vary slowly over mesoscopic times and length scales. Being even in the apolar layer normal ${{{{{{{\bf{N}}}}}}}}\left({{{{{{{\bf{r}}}}}}}},\, t\right)$, the phase does not possess the same ambiguity as Φ. A change of *ϕ* by 2*π* indicates that the layer displacement *u* has increased by a full layer thickness, which necessitates the existence of an additional layer in this mesoscopic model.

To account for the apolar symmetry of the layer normal, the smectic tensorial order parameter ${{{{{{{\bf{E}}}}}}}}\left({{{{{{{\bf{r}}}}}}}},\, t\right)$ must contain the dyadic square of **N**, making **E** symmetric. Furthermore, the absence of preferential directions within planar layers indicates local rotations about **N** are arbitrary. A traceless order parameter ensures linear terms do not contribute to the bulk free energy. Based on these considerations, we propose the uniaxial complex-tensorial smectic order parameter

1 $${{{{{{{\bf{E}}}}}}}}\left({{{{{{{\bf{r}}}}}}}},\, t\right)=\psi \left({{{{{{{\bf{N}}}}}}}}\otimes {{{{{{{\bf{N}}}}}}}}-\frac{{{{{{{{\boldsymbol{\delta }}}}}}}}}{d}\right).$$

The scalar order parameter $\psi \left({{{{{{{\bf{r}}}}}}}},\, t\right)=\left|\psi \right|{e}^{i\phi }\in {\mathbb{C}}$ is the largest eigenvalue of **E** (with $\left|\psi \right|\in {\mathbb{R}}$ and *ϕ* ∈ *S*^1^) and the layer normal ${{{{{{{\bf{N}}}}}}}}\left({{{{{{{\bf{r}}}}}}}},\, t\right)\in {{\mathbb{R}}}^{d}$ is the associated eigenvector. The tensor describes mesoscopic variations of lamellar order in the hydrodynamic limit, treating lengths on the scale of individual layers as microscopic. It is symmetric, traceless and globally gauge invariant (under **E** → *e*^*iθ*^**E** for arbitrary *θ*); furthermore, it allows **N** → − **N** and, by using *ϕ*, avoids interpretation issues stemming from the double-valued nature of Φ. This limits its consideration only to mesoscopic variations in layer displacement. While a generic second-rank tensor has *d*^2^ elements, these constraints ensure **E** only possesses *d* + 1 degrees of freedom, representing the extent of layering, layer displacement, and unit layer normal. As in nematic **Q**-theory, local biaxiality is possible in **E** in 3D but biaxiality at equilibrium requires higher order terms in the bulk free energy^64^ (see Methods). In describing these slowly varying aspects of a lamellar system without reference to any specific form of the microscopic density variation, **E** acts as a mesoscopic order parameter that is not based on or limited to any particular molecular details or assumptions about the layer structure.

Though smectics and other lamellae have been modeled from many perspectives^12,33,65,66^, we consider a Landau free energy expansion. The total free energy density *f* is the sum of bulk and two deformation (compression and curvature) terms. All contributions to the free energy must be real, requiring pairings of **E** and its complex conjugate E^*^. Furthermore, the free energy should not depend on **E** in a manner that is equivalent to a direct dependence on the phase, since it can be globally shifted.

### Bulk

Since **E** is traceless, the bulk smectic free energy density can be written

2 $${f}^{{{{{{{{\rm{bulk}}}}}}}}}=\frac{A}{2}{E}_{ij}{E}_{ji}^{*}+\frac{C}{4}{\left({E}_{ij}{E}_{ji}^{*}\right)}^{2}+\ldots$$

where *C* > 0, and Einstein summation convention is adopted. Lamellar order is established when *A* < 0, but the fluid is isotropic when *A* > 0. The bulk free energy does not depend on phase or layer normal, but only on $\left|\psi \right|$. This form is consistent with scalar-based bulk free energies^36,37,67^ (see Methods). In the mean-field limit, Eq. (2) predicts a second order phase transition.

### Compression

Lamellae possess two deformation modes: (i) compression, and (ii) curvature of the layers. We consider first compression free energies, which involve derivatives of the tensor order parameter. The simplest such term is ${E}_{ij,k}{E}_{ij,k}^{*}$, where *k* denotes the direction of the gradient. Additional real terms could be constructed through combinations of similar forms, which would allow different deformation modes to possess differing elastic modulii. For clarity, we make a one-constant approximation

3 $${f}^{{{{{{{{\rm{el}}}}}}}}}={b}_{1}{E}_{ij,k}{E}_{ji,k}^{*},$$

where *b*_1_ is a layer compression elastic constant. Equation (3) accommodates first order distortions of the layer normal, as well as contributions due to gradients of the complex amplitude *ψ*. In a vector-based model, defects would create topological singularities in the layer normal field, making gradient terms in Eq. (3) irregular; however, **E** regularizes the singularities and Eq. (3) is continuous.

### Curvature

Distortions from uniformly aligned layers come with a free energy cost, akin to a membrane curvature free energy density. We again make a one-constant approximation and keep only the simplest term

4 $${f}^{{{{{{{{\rm{curv}}}}}}}}}={b}_{2}{E}_{ij,kk}{E}_{ji,\ell \ell }^{*},$$

where *b*_2_ is a bending modulus.

By inserting the eigenvalue and associated eigenvector via Eq. (1) into the free energy contributions (Eqs. (2)–(4)), one can directly compare **E**-theory to existing models of smectics (Methods). When the lamellae phase is free of deformations, minimizing the free energy produces the equilibrium value

5 $${\left|\psi \right|}^{{{{{{{{\rm{eq}}}}}}}}}=\left\{\begin{array}{ll}\sqrt{-\frac{A}{C\sigma }}\quad &{{{{{{{\rm{if}}}}}}}}\,A\,\le \,0\\ 0\quad &{{{{{{{\rm{otherwise}}}}}}}}\end{array}\right.,$$

where $\sigma=\left(d-1\right)/d$, which is in agreement with complex scalar Landau models^36,37^. In our model, **E** is a hydrodynamic-scale field that does not involve layer spacing, so imposing an equilibrium wave number would require that covariant derivatives replace gradients^67–69^. At equilibrium, the free energy density is ${f}^{{{{{{{{\rm{eq}}}}}}}}}=-\frac{\sigma }{2}\frac{{A}^{2}}{C}\left(1-\frac{\sigma }{2}\right)$. In contrast to nematics, the free energy possesses two material length scales: (i) coherence length $\xi=\sqrt{{b}_{1}/A}$ and (ii) penetration depth $\lambda=\sqrt{{b}_{2}/{b}_{1}}$. The coherence length *ξ* characterizes the defect core size and the ratio of *κ* = *λ*/*ξ* is a Ginzburg parameter. As in superconductors, $\kappa < 1/\sqrt{2}$ is a type-I system, while $\kappa > 1/\sqrt{2}$ is type-II^20^. We also take the strong anchoring limit by fixing **E** at solid surfaces.

**Fig. 2:**
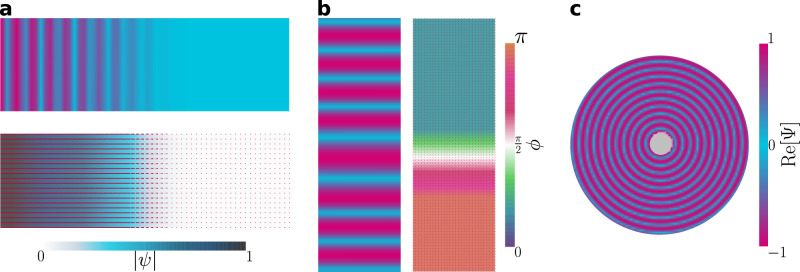
Smectic layer configurations corresponding to changes to a single aspect of the order parameter. Smectic layers configurations for a variety of confining geometries at steady-state. *C* = 2, *κ*^2^ = 0.6 and *A* = − 1, unless otherwise stated. **a** Modeling a temperature gradient in a channel, *A* increases linearly from left to right passing through *A* = 0 at the center. Layering disappears as the smectic passes into the isotropic phase. (**a**; top) ${{{{{{{\rm{Re}}}}}}}}\left[{{\Psi }}\right]$; (**a**; bottom) $\left|\psi \right|$ with **N** overlaid. **b** Local dilation of layers, through linearly decreasing *ϕ* from *π* to 0 over a region of a channel. (left) ${{{{{{{\rm{Re}}}}}}}}\left[{{\Psi }}\right]$ and (right) *ϕ* with **N** overlaid. **c** Layers bend without compression/dilation due to homeotropic anchoring of the layer normal to the walls of a confining annulus.

Proven numerical schemes exist for minimizing the free energy of real **Q** tensors^70^. The numerical difficulty lies in extending the methodology to allow for complex tensor elements. We employ a gradient descent time evolution of ${{{{{{{\bf{E}}}}}}}}\left({{{{{{{\bf{r}}}}}}}},\, t\right)$ in 2D (see Methods^71^). Defining the total free energy $F\left(t\right)=\int{d}^{d}{{{{{{{\bf{r}}}}}}}}\,f\left({{{{{{{\bf{E}}}}}}}}\left({{{{{{{\bf{r}}}}}}}},\, t\right),\, {{{{{{{\boldsymbol{\nabla }}}}}}}}{{{{{{{\bf{E}}}}}}}}\left({{{{{{{\bf{r}}}}}}}},\, t\right)\right)$, we adopt a time-dependent Ginzburg-Landau model

6 $$\mu \frac{\partial {E}_{ij}}{\partial t}=-\frac{\delta F}{\delta {E}_{ij}^{*}}+{{{\Lambda }}}_{ij},$$

where *μ* is a mobility coefficient and **Λ** constrains **E** to be traceless and normal (see Methods). It should be stressed that ${{{{{{{\bf{E}}}}}}}}\left({{{{{{{\bf{r}}}}}}}},\, t\right)$ is the sole subject of all calculations—the complex amplitude $\psi \left({{{{{{{\bf{r}}}}}}}},\, t\right)$ and layer normal ${{{{{{{\bf{N}}}}}}}}\left({{{{{{{\bf{r}}}}}}}},\, t\right)$ are only found *ex post facto*. Both $\left|\psi \right|$ and *ϕ* are calculated directly from contractions of **E** with itself, while **N** is found via eigen-decomposition (see Methods). Defects are identified from the **N** and *ϕ* fields (see Methods). While our approach circumvents the ambiguity of Ψ as a double-valued function ${{{{{{{\rm{Re}}}}}}}}\left[{{\Psi }}\right]\pm i {{{{{\mathrm{Im}}}}}}\,\left[{{\Psi }}\right]$^15,26^, the post-processing visualizations based on ${{{{{{{\rm{Re}}}}}}}}\left[{{\Psi }}\right]$ introduce aberrations that **E** itself does not possess. It is clear that these aberrations (see Fig. 1c–e, Fig. 2 and Methods) are not visible in the $\left|\psi \right|$ or *ϕ* fields, appearing only as a result of reintroducing the microscopic layer structure.

## Discussion

**Fig. 3:**
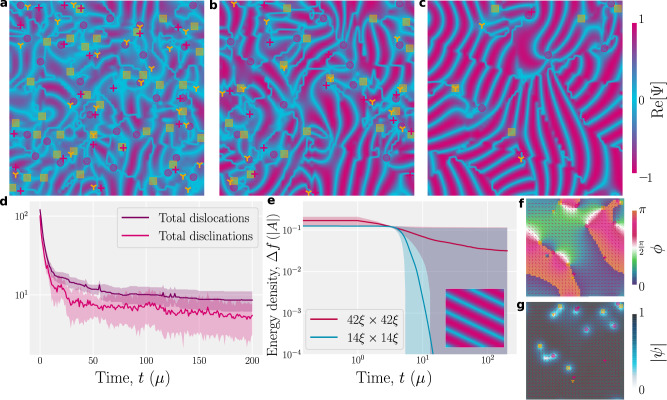
Deep quench of the simple smectic results in a kinetically arrested state, with multiple defects. Simulations initialized from an isotropic state ($\left|\psi \right|\simeq 0$ and random **N** and *ϕ*) with *A* = − 1 (lamellar state), *C* = 2, *κ*^2^ = 0.5 and periodic boundary conditions. **a**–**c** Snapshots of ${{{{{{{\rm{Re}}}}}}}}\left[{{\Psi }}\right]$ in a system size of 42*ξ* × 42*ξ* at times **a** *t* = 2*μ*; **b** 5*μ*; and **c** long-time limit kinetically arrested state (snapshot at time *t* = 20*μ*). Pink crosses (yellow trilaterals) mark + 1/2 (−1/2) disclinations. Edge dislocations with winding number ± 1 denoted by pink circles and yellow squares. **d** Temporal dependence of the average defect density. Shading represents the standard deviation. **e** Temporal dependence of the free energy density, Δ*f* = *f* − *f*^eq^, for small (14*ξ* × 14*ξ*) and large (42*ξ* × 42*ξ*) systems. Shading represents the standard deviation. (inset) Steady state for 14*ξ* × 14*ξ*. **f** Snapshot of *ϕ*(**r**; *t*) corresponding to **c**. **g** The corresponding ∣*ψ*(**r**, *t*)∣ field.

The ability of **E**-theory to model simple smectics is probed by simulating a variety of confining geometries (Fig. 2). We first demonstrate that individual aspects of the order parameter can vary independently. In a long slit with **N** strongly anchored parallel to the walls, a temperature gradient is modeled via a linear increase of the bulk free energy (Eq. (2)) parameter *A* from −1 to 1 (Fig. 2a). This causes $\left|\psi \right|\left({{{{{{{\bf{r}}}}}}}}\right)$ to decrease, going from an ordered lamellar state on the left to the isotropic state with $\left|\psi \right |=0$ on the right, in agreement with Eq. (5), and without variation of *ϕ* or **N**. After minimization of the free energy, ${{{{{{{\rm{Re}}}}}}}}\left[{{\Psi }}\right]$ visualizes the lamellar structure and the layer normal reflects the direction of layering (Fig. 2a). Similarly, the phase $\phi \left({{{{{{{\bf{r}}}}}}}}\right)$ can be varied within a long slit without variation of $\left|\psi \right|$ or **N** by linearly increasing the phase over a narrow region of the channel walls (Fig. 2b). Again, **N** is strongly anchored parallel to the walls, which causes compressional distortion—the layers to dilate, as seen from ${{{{{{{\rm{Re}}}}}}}}\left[{{\Psi }}\right]$. The final pure type of deformation is distorting ${{{{{{{\bf{N}}}}}}}}\left({{{{{{{\bf{r}}}}}}}}\right)$. To create this deformation, the smectic is confined within an annulus with strong homeotropic anchoring of the layer normal (Fig. 2c). This geometry produces pure bend distortion with no compression that changes *ϕ*. Not all geometries allow the aspects of the order parameter to vary independently, as accomplished by Fig. 2a-c; in general, we expect some interplay between deformation modes as in Fig. 3.

There are two classes of defects in smectic systems (Fig. 1). The first relates to singularities in the layer normal **N**, in which **N** rotates by 2*πm*_**N**_, we show this occurring in two ways: (i) A ${m_{\vec{{{\mathbf{N}}}}}=+1/2}$ disclination with a 180-degree folding of smectic layers around the defect core (Fig. 1b; left) or a ${m_{\vec{{{\mathbf{N}}}}}=-1/2}$ disclination in which the layers exhibit a trifold symmetry (Fig. 1b; center). (ii) An irregularity in the layer structure, which represents an insertion/deletion of layers at a point (Fig. 1b; right). To identify and measure the respective charges of these, we measure winding numbers using a closed contour integral (Methods). To explore the capacity of this model to describe smectic defects, consider a circular confining domain with boundary conditions requiring a single +1/2 disclination (Fig. 1c). After minimization of the free energy, ${{{{{{{\rm{Re}}}}}}}}\left[{{\Psi }}\right]$ depicts the lamellar structure around the disclination (Fig. 1f). Since **N** is the layer normal, it mirrors the layers (Fig. 1f, i). The lamellar structure exhibits the expected symmetries of a +1/2 disclination and deformations are primarily bend on one side of the defect and splay on the other^72^. The lamellae are highly ordered away from the defect with $\left|\psi \right|\to {\left|\psi \right|}^{{{{{{{{\rm{eq}}}}}}}}}$. However, $\left|\psi \right|\to 0$ in the defect core (Fig. 1f), verifying that **E**-theory permits a finite sized defect core. Crucially, **E** remains continuous at the center of the defect, regularizing the singularity in **N**. The deformations are principally curvature distortions, rather than compression, which is reflected in a constant phase everywhere in the vicinity of the disclination (Fig. 1i). We find no evidence of any artificial order parameter melting where *ϕ*→−*ϕ*, suggesting that the non-physical line tension and associated free energy penalty observed in simulations of folded layers using two-dimensional scalar theories^26^ is absent (Supplementary Fig. 1). The situation is analogous for a −1/2 disclination (Fig. 1d): The layers are visualized by ${{{{{{{\rm{Re}}}}}}}}\left[{{\Psi }}\right]$ (Fig. 1d), with perpendicular layer normals (Fig. 1g, j). The defect core is again seen to be locally disordered with no variation in phase, indicating negligible compression. In both ±1/2 disclinations, the free energy density is largest in the immediate vicinity of the cores (Supplementary Fig. 2). Not only is *f* ^bulk^ non-constant only at the core, but the deformation energy densities are strongly localized^72^. In addition to half-integer disclinations, **E**-theory has the capacity to simulate +1 disclinations that have been observed in transition to pairs of separated +1/2 disclinations in circular confinement with strong homeotropic anchoring (Supplementary Fig. 3).

In addition to disclinations, which are present in both nematic and lamellar phases, edge dislocations are unique to materials with broken translational symmetry. While the phase *ϕ* is physically invariant to a global shift, it is set to vary linearly at the circular confining boundaries as *ϕ* = *θ*/2 for polar angle *θ* (Fig. 1e). This results in a dislocation: An extra layer is generated on the bottom half of Fig. 1e. While the lamellar order $\left|\psi \right|$ still decreases in the defect core (Fig. 1h) and the order parameter variations are still localized around the core (Supplementary Fig. 2), the phase changes by 2*π* around the dislocation (Fig. 1k). The occurrence of independent disclinations (Fig. 1c, d) and dislocations (Fig. 1e) highlights a strength of **E**-theory: since theories of *ϕ* alone cannot model independent disclinations and models that simulate **Q** near the nematic-smectic transition cannot replicate dislocations. While disclinations and dislocations are considered separately in Fig. 1, they can co-reside in a single defect.

We now consider the role of defects in lamellar states evolving to equilibrium by simulating 2D systems with a deep quench from the isotropic to lamellar state, and periodic boundary conditions (Fig. 3). At first, the system is disordered (Fig. 3a), but relaxes through defect annihilation (Fig. 3b) to form many locally ordered domains (Fig. 3c). However, even at the longest times, the system remains disordered on mesoscopic scales: It is kinetically arrested into a glassy configuration^73^ with a non-zero number of defects (Fig. 3d). Within this glassy state are domains for which **N** is rotated by *π*/2 (Fig. 3c, g), which is allowed by Eq. (1) in 2D when accompanied by *ϕ*→*ϕ*±*π* (Fig. 3f) and correspond to the bridge-type line boundaries observed in 2D colloidal smectics^34^.

To clarify this pinning of long-lived non-equilibrium structures, we compare simulations of large and small systems. While the small system routinely relaxes to the fully ordered lamellar state (Fig. 3e; inset and Supplementary Movie 1) with $\mathop{\lim }\nolimits_{t\to \infty }\left|\psi \right|\to {\left|\psi \right|}^{{{{{{{{\rm{eq}}}}}}}}}$, the large system never reaches the global equilibrium (Fig. 3c). Correspondingly, the free energy of the small system rapidly approaches *f*^ eq^, the equilibrium defect free value; whereas, the large system is inevitably trapped away from equilibrium (Fig. 3e). Snapshots and associated videos show that both disclinations and edge dislocations are pinned^74^ (Fig. 3f, g and Supplementary Movie 2). This highlights the importance of defects in lamellar ordering kinetics and the challenge posed for lamellar self-assembly^10–12,32,33^. In contrast to the continual relaxation dynamics through annihilation in nematic liquid crystals^75,76^, the kinetic arrest of coarsening and long-lived domains are associated with pinned defects (Fig. 3e)^73,77^. This implies an energy barrier associated with the sliding of dislocations with respect to the lamellar structure. This indicates the possibility of non-zero Peierls-Nabarro energy barriers^26,48^, further validating of the **E** formulation.

**Fig. 4:**
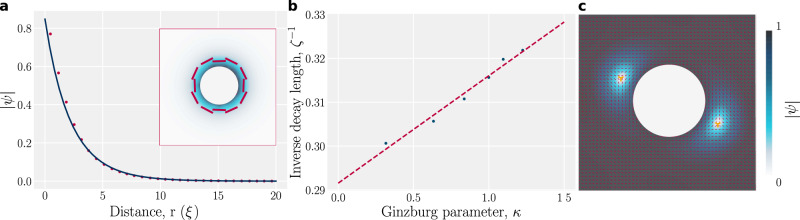
Circular inclusions induce local smectic order and set the topological charge of the domain. **a** A circular inclusion embedded in a bulk isotropic phase showing boundary-induced local lamellar ordering (radius *R* = 5*ξ*, *A* = 0.1 (isotropic state), *C* = 2, *κ*^2^ = 0.5, anchoring: *ψ* = *e*^*iπ*/2^, **N** parallel to the boundary). Exponential decay of lamellar order $\left|\psi \right|$ with distance from the surface *r*. (inset) Snapshot of $\left|\psi \right|$ with layer normal shown in red. **b** Inverse of the exponential decay length *ζ* as a function of Ginzburg parameter *κ* for the isotropic phase confined between planar walls. **c** Plot of $\left|\psi \right|$ in the vicinity of an inclusion (same parameters as **a** except *R* = 4*ξ* and *A* = −1 (lamellar state)). The system is initialized near isotropic ($\left|\psi \right|\sim 0.2$).

The presence of inclusions embedded within the lamellar material can act to locally order layers or to induce additional defects. We evaluate boundary-induced lamellar ordering within an isotropic fluid (*A* > 0), due to strong anchoring to a circular inclusion (Fig. 4a). An inclusion with strong planar anchoring of **N** and *ψ* = *e*^*iπ*/2^ locally layers the smectic but the ordering rapidly decays (Fig. 4a). By fitting an exponential to $\left|\psi \right|$ in a channel geometry, we extract the decay length *ζ* (Fig. 4b). We see that the decay length varies inversely with the Ginzburg parameter *κ*, indicating *ζ* varies linearly with lamellar coherence length *ξ*. While strong anchoring locally orders the isotropic phase, it induces a pair of defects in the lamellar phase (Supplementary Movie 6). The steady-state can be seen from the layer normal field (Fig. 4c) or directly from the qualitative layer visualization via ${{{{{{{\rm{Re}}}}}}}}\left[{{\Psi }}\right]$ (Supplementary Fig. 4a). The topological charge of the circular inclusion is neutralized by the two −1/2 disclinations on opposite poles of the inclusion. The resemblance to a nematic system^78^ follows from their shared *π*-rotational symmetry. Outside of the defect cores, the smectic remains well ordered and the deformation free energy contributions are localized around the inclusion (Supplementary Fig. 5). This demonstrates the **E**-formalism can be employed for nontrivial geometries.

**Fig. 5:**
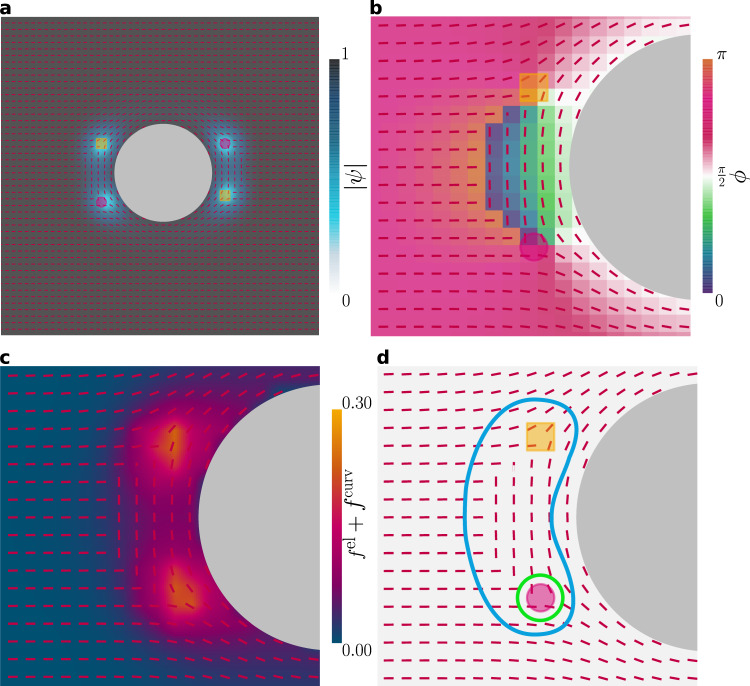
Half-charge disclinations can split into quarter-charge points at the ends of a grain boundary. Same as Fig. 4, except the phase field is initialized to have one value at the surface of the inclusion (*ϕ*^surface^ = *π*/2) but a significantly different value in the bulk *ϕ*^bulk^ ≃ 3*π*/4. This results in a grain boundary, with endpoints of −1/4 topological charge. With **N** overlaid on each plot. **a** Modulus $\left|\psi \right|$ field. **b** Phase *ϕ* field. Zoom showing that the layer normal direction turns by *π*/2 across the grain boundary. **c** Elastic free energy density *f*^ el^ + *f*^ curv^ normalized by $\left|A\right|$. **d** Layer normal **N** with both quarter-charge defects marked. By taking a contour around both defects (blue path) the topological charge is −1/2 (by Eq. (25)). However circling an individual end-point defect (green path) results in a topological charge of −1/4.

In strongly confined two-dimensional smectics composed of colloidal silica rods, it has recently been reported that half-charge ±1/2 disclinations can expand into grain-boundary lines capped by pairs of end-point ±1/4 charge defects^34^. These quarter-charge defects have been numerically reproduced using microscopic density functional theory^34^ and explored using extensive Monte Carlo simulations^35,79^ but are yet to be described by a mesoscopic continuum theory. To demonstrate the complex tensor **E** description has the capacity to simulate such defect structures, a circular inclusion is once again embedded in a simple smectic (as in Fig. 4), but initialized with a significant difference of phase *ϕ* at the inclusion surface compared to the bulk (Fig. 5b). This causes the −1/2 disclinations seen in Fig. 4 to each split into two −1/4 charge end-point defects capping a bridging line boundary. Such behavior is not expected nor observed in nematic liquid crystals, and does not arise in **Q**-tensor theories of nematics but can be reproduced by the **E**-tensor formalism for lamellar fluids. Both of the new quarter-charge end points are seen to possess disordered defect cores in which $\left|\psi \right|\to 0$ (Fig. 5a). The phase *ϕ* changes discontinuously across the line boundary and a *π*/2 misalignment of the layer normal occurs (Fig. 5b). This can also be seen in the plots of ${{{{{{{\rm{Re}}}}}}}}\left[{{\Psi }}\right]$ (Supplementary Fig. 4b). However, if the phase is integrated around the whole structure, rather than through the line boundary, the winding number of the phase of the structure is zero (Fig. 5d).

While the end-point defect cores have a free energy cost, no free energy cost is associated with the line boundary (Fig. 5c). In 2D, the eigenvalues of the traceless **E** differ by only a sign. This reflects the fact that a 2D smectic system can be described equally well by the layer normal **N** or the perpendicular layer tangent. Indeed, the layer normal and layer tangent can be swapped so long as *ϕ* → *ϕ* + *π*, thereby exchanging the signs of the eigenvalues. This suggests that such line boundaries are only possible in 2D, since the eigenvalue associated with the layer normal in 3D is distinct from the two degenerate eigenvalues associated with the pair of in-plane unit vectors.

**Fig. 6:**
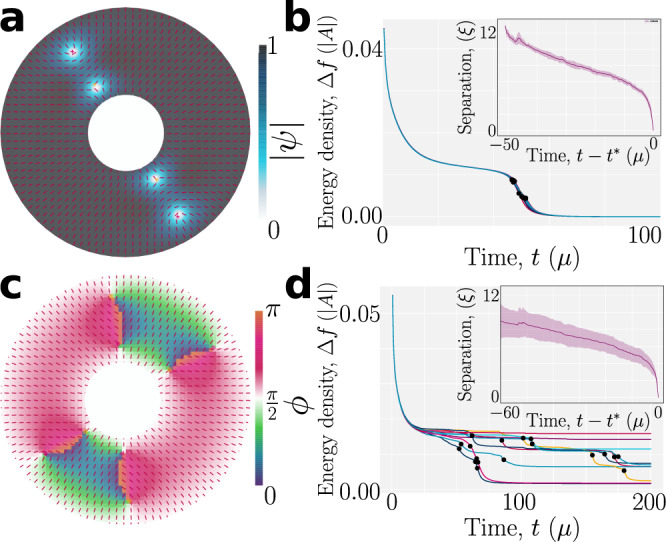
Annihilation of quarter- and half-charged disclinations exhibit different relaxation dynamics. Systems are initialized with (**a**, **b**) or without (**c**, **d**) a phase gradient between the bulk and boundary in annular confinements, leading to either two pairs of half-charged disclinations (**a**) or four pairs of quarter-charged disclinations (**c**). All parameters are as in Fig. 5. **a** The modulus $\left|\psi \right|$ for a system initialized to produce two pairs of half-charged disclinations at time *t* = 15*μ*. **b** Temporal dependence of the free energy density, Δ*f* = *f* − *f*^eq^ for repeated simulations with the **a** initialization. Black circles mark defect annihilation events. (inset) Temporal evolution of defect separation for each pair relative to t^*^, the pair annihilation time. Shading represents the standard deviation. **c** The *ϕ* field for a system initialized to produce four pairs of quarter-charged disclinations at time *t* = 15 *μ*. **d** Same as **b** but for quarter-charge annihilation dynamics.

Simulating the relaxation of a strongly anchored system in a annular confinements allows us to analyze the differing relaxation dynamics of both half- and quarter-charged disclinations. Systems initialized with no significant difference of phase *ϕ* at the boundaries compared to the bulk see formation of half-charged disclination defects (Fig. 6a, b). Annihilation dynamics are consistent across repeated initializations with noise in *ψ*, *ϕ* and **N** (Fig. 6b; inset). However, systems forming pairs of quarter-charge defect complexes (Fig. 6c, d) undergo much less consistent annihilation under equally varied initial conditions (Fig. 6d; inset), with 65% remaining after long times (*t* > 150*μ*). This is consistent with the dynamics observed in Fig. 3. By comparing the free energy density at times 5*μ* before and after annihilation events, the mean free energy per unit area change for a pair of half-charge defects is (5.24 ± 0.04) × 10^−3^∣*A*∣ compared to (3.8 ± 0.5) × 10^−3^∣*A*∣. We note that quarter-charge defects are more costly per unit absolute topological charge than half-charge defects in these simulations, possibly accounting for their scarce creation in systems missing an enforced initial phase gradient of *ϕ*.

We have proposed a complex, symmetric, traceless, globally gauge invariant, normal, uniaxial tensorial order parameter ${{{{{{{\bf{E}}}}}}}}\left({{{{{{{\bf{r}}}}}}}},\, t\right)$ for describing simple smectic phases at mesoscopic scales. As a second-rank tensor, **E** encodes the apolar nature of the layer normal in an arbitrary reference frame and circumvents the ambiguity of using a complex scalar order parameter. It does so without resorting to a microscopic approach, such as density functional theory^34^, real-valued density variation^26,31^ or particle-based simulations^35,79^, which also bypass such ambiguities but at a higher computational cost. While such microscopic models can simulate microscopic structure of individual layers, a numerically amenable framework for simulating the mesoscopic variations is advantageous for modeling configurations and dynamics at large length and time scales. By conjoining local layer orientation and the extent of ordering into a single mathematical object, the **E** tensor is a mesoscopic description that can reproduce both disclination and dislocation defects with finite defect cores.

Though individual singularities can be elegantly analytically handled through local branch cuts, the tensor order parameter description globally eliminates this ambiguity in a numerically pragmatic manner. Akin to the nematic **Q** tensor, this has the numerical advantage of avoiding point singularities. In the presence of disclinations, the layer normal **N** is a singular vector, while the phase *ϕ* is singular about dislocations—the tensor **E** regularizes the singularities and remains continuous at the center of defects. In this paper, we have restricted our consideration to a “simple” smectic (purely lamellar) idealization that does not require a well-defined nematic director as a prerequisite of spontaneous lamellar symmetry breaking. Liquid crystalline phases such as smectic-A or -C are composed of anisotropic molecules that also exhibit nematic ordering, and simulating these will require coupling of **E** to **Q**, which is conceptually straightforward^36,80^.

Additionally, the **E**-formalism should be extended to consider more complex systems. Smectic textures in three dimensions can be complex^81^, appealing^82^ and difficult to model^31^. The tensor theory could be extended to simulate patterned defect arrays through programmable photoalignment^83^, electrically reversible templating^84^ and micropatterned substrates^18^. Much like the introduction of **Q** helped expand the possibilities for numerical modeling of nematics, we expect this framework to be advantageous for simulating colloidal smectics^82,85–90^, smectic-isotropic interfaces^91,92^, smectic-smectic emulsions^93^, smectics in contact with active material^94^ and swimming bacteria in smectics^95^.

## Methods

### Numerical methods

We employ a gradient descent evolution of ${{{{{{{\bf{E}}}}}}}}\left({{{{{{{\bf{r}}}}}}}};t\right)$. This is to say that ${{{{{{{\bf{E}}}}}}}}\left({{{{{{{\bf{r}}}}}}}};t\right)$ obeys a time-dependent Ginzburg-Landau model, which follows the steepest decrease in global free energy under the constraints that it remain traceless and uniaxial. This is described by Eq. (6) in the main text and Eq. (16) in Methods.

The total free energy density is the sum of Eq. (2)-(4),

7 $$f	={f}^{{{{{{{{\rm{bulk}}}}}}}}}+{f}^{{{{{{{{\rm{el}}}}}}}}}+{f}^{{{{{{{{\rm{curv}}}}}}}}}\\ 	=\frac{A}{2}{E}_{ij}{E}_{ji}^{*}+\frac{C}{4}{\left({E}_{ij}{E}_{ji}^{*}\right)}^{2}+{b}_{1}{E}_{ij,k}{E}_{ji,k}^{*}+{b}_{2}{E}_{ij,kk}{E}_{ji,\ell \ell }^{*}.$$

At each timestep, we use the instantaneous free energy density to calculate the right hand side of Eq. (6). The Lagrange multipliers can be found directly but the functional derivative term involves spatial derivatives. The system is discretised in space on a square grid of step size Δ*x*. We employ a two-step Adams-Bashforth method to calculate **E** at the next timestep, using a discrete time step of Δ*t*. This is iterated for *T* time steps. For Fig. 2, 3, Δ*x* = 0.7*ξ*, Δ*t* = 0.001*μ*. For Fig. 2, *T* = 15000, which amounts to a total simulation time of 15*μ*; for Fig. 1, *T* = 20,000 (or 20*μ*); and for Fig. 3*T* = 200,000. In Fig. 4a, Δ*x* = 0.8*ξ*, Δ*t* = 0.001*μ* and *T* = 100,000; Δ*x* = 0.5*ξ*, Δ*t* = 0.0001*μ* and *T* = 2000,000 in Fig. 4b and Δ*x* = 0.4*ξ*, Δ*t* = 0.0001*μ* and *T* = 100,000 in Fig. 4c. In Fig. 5, Δ*x* = 0.8*ξ*, Δ*t* = 0.001*μ* and *T* = 10,000. We note that these Δ*x* and Δ*t* are chosen case by case and all choices are robust under reducing the chosen values.

We employ specific boundary and initial conditions for each system considered, as discussed in the main text. To initialize the system, we specify **E** at each point in space, which we do by setting a local *ϕ*, $\left|\psi \right|$ and **N**. These are then converted to **E** through the definition Eq. (1).

After initialization, we directly simulate **E**, without any reference to *ϕ*, $\left|\psi \right|$ and **N**. Only after the simulations have completed are *ϕ*, $\left|\psi \right|$ and **N** found from the resulting **E**. The complex scalar order parameter *ψ* corresponds to one eigenvalue and **N** is the associated eigenvalue. Due to the tracelessness of **E**, the pair of eigenvalues contain the same information and differ only by their sign in 2D. In 3D, the pair of in-plane eigenvectors (i.e., the pair that are orthogonal to the layer normal **N**) must be interchangeable and so their associated eigenvalues are degenerate, while the third is constrained to be proportional by the tracelessness condition.

As complex eigen-decomposition is numerically imprecise, we find the following method more reliable. The scalar order parameter $\left|\psi \right|$ is found directly by contracting the complex tensor with its complex conjugate, ${{{{{{{\bf{E}}}}}}}}:{{{{{{{{\bf{E}}}}}}}}}^{*}=\sigma {\left|\psi \right|}^{2}$. Explicitly, $\left|\psi \right |=\sqrt{{E}_{ij}{E}_{ji}^{*}/\sigma }$. Likewise, the phase *ϕ* is found by contracting the complex tensor with itself ${{{{{{{\bf{E}}}}}}}}:{{{{{{{\bf{E}}}}}}}}=\sigma {\left|\psi \right|}^{2}{e}^{2i\phi }$, once $\left|\psi \right|$ is known. Explicitly, $\phi=(-i/2)\arg [{E}_{ij}{E}_{ji}/(\sigma {\left|\psi \right|}^{2})]$, which returns *ϕ* ∈ [−*π*, *π*). Finally, to determine the layer normal direction **N**, we apply eigen-decomposition to the real tensor *e*^−*ϕ*^**E**, recovering a pair of real eigenvectors. In the standard case, the eigenvector corresponding to the positive eigenvalue is selected as **N**. However in systems with boundary conditions enforcing a phase discontinuity, such as in Fig. 1c, we must enforce the choice of eigenvalue along each boundary and promulgate that numerical decision though the system. This decision prevents an artifical discontinuity in the layer normal along an arbitrary line due to a phase shift of *π* swapping the signs of the eigenvalues, and hence the choice of corresponding eigenvector. This argument is avoided in 3D as the eigenvalues are *ψ*, −*ψ*/2, −*ψ*/2; so the eigenvalue with the largest modulus should always be chosen.

### Lagrange multipliers

We enforced the fact that **E** be traceless, uniaxial and a normal operator, which commutes with its own adjoint, in the numerics. This is essential for ensuring **E** maintains real eigenvectors and allows us to interpret the **E** after the simulations. These two conditions can be written as

8 $${g}_{1}({{{{{{{\bf{E}}}}}}}})={E}_{ii}=0$$

9 $${g}_{2}({{{{{{{\bf{E}}}}}}}})=\det \left([{{{{{{{\bf{E}}}}}}}},\, {{{{{{{{\bf{E}}}}}}}}}^{*}]\right)=0,$$

where [*A*, *B*] = *AB* − *BA* denotes the commutator. Using the Cayley–Hamilton Theorem and noting *g*_2_ can be rewritten

10 $${g}_{2}({{{{{{{\bf{E}}}}}}}})=-\frac{1}{2}{{{{{{{\rm{tr}}}}}}}}\left({[{{{{{{{\bf{E}}}}}}}},\, {{{{{{{{\bf{E}}}}}}}}}^{*}]}^{2}\right).$$

Decomposing *g*_1_ into real and imaginary parts, ${g}_{1a}={{{{{{{\rm{Re}}}}}}}}\left[{g}_{1}\right]$ and ${g}_{1b}={{{{{{{\rm{Im}}}}}}}}\left[{g}_{1}\right]$, we introduce three real Lagrange multipliers, *λ*_1*a*_, *λ*_1*b*_ and *λ*_2_, defined as

11 $${\lambda }_{1a}={{{{{{{\rm{Re}}}}}}}}\left[\frac{1}{d}\left(\frac{\delta F}{\delta {E}_{ii}}-{\lambda }_{2}\frac{\partial {g}_{2}}{\partial {E}_{ii}}\right)\right]$$

12 $${\lambda }_{1b}={{{{{{{\rm{Im}}}}}}}}\left[\frac{1}{d}\left(\frac{\delta F}{\delta {E}_{ii}}-{\lambda }_{2}\frac{\partial {g}_{2}}{\partial {E}_{ii}}\right)\right]$$

13 $${\lambda }_{2}=\frac{{c}_{1}}{{c}_{2}}.$$

Where *c*_1_ and *c*_2_ are

14 $${c}_{1}	=- \frac{\delta F}{\delta {Y}_{ik}}{X}_{kj}{Y}_{jp}{X}_{pi}-{Y}_{ik}\frac{\delta F}{\delta {X}_{kj}}{Y}_{jp}{X}_{pi}-{Y}_{ik}{X}_{kj}\frac{\delta F}{\delta {Y}_{jp}}{X}_{pi}-{Y}_{ik}{X}_{kj}{Y}_{jp}\frac{\delta F}{\delta {X}_{pi}}\\ 	+\frac{\delta F}{\delta {Y}_{ik}}{X}_{kj}{X}_{jp}{Y}_{pi}+{Y}_{ik}\frac{\delta F}{\delta {X}_{kj}}{X}_{jp}{Y}_{pi}+{Y}_{ik}{X}_{kj}\frac{\delta F}{\delta {X}_{jp}}{Y}_{pi}+{Y}_{ik}{X}_{kj}{X}_{jp}\frac{\delta F}{\delta {Y}_{pi}}\\ 	+\frac{\delta F}{\delta {X}_{ik}}{Y}_{kj}{Y}_{jp}{X}_{pi}+{X}_{ik}\frac{\delta F}{\delta {Y}_{kj}}{Y}_{jp}{X}_{pi}+{X}_{ik}{Y}_{kj}\frac{\delta F}{\delta {Y}_{jp}}{X}_{pi}+{X}_{ik}{Y}_{kj}{Y}_{jp}\frac{\delta F}{\delta {X}_{pi}}\\ 	 -\frac{\delta F}{\delta {X}_{ik}}{Y}_{kj}{X}_{jp}{Y}_{pi}-{X}_{ik}\frac{\delta F}{\delta {Y}_{kj}}{X}_{jp}{Y}_{pi}-{X}_{ik}{Y}_{kj}\frac{\delta F}{\delta {X}_{jp}}{Y}_{pi}-{X}_{ik}{Y}_{kj}{X}_{jp}\frac{\delta F}{\delta {Y}_{pi}}$$

15 $${c}_{2}	=-\frac{\partial {g}_{2}}{\partial {Y}_{ik}}{X}_{kj}{Y}_{jp}{X}_{pi}-{Y}_{ik}\frac{\partial {g}_{2}}{\partial {X}_{kj}}{Y}_{jp}{X}_{pi}-{Y}_{ik}{X}_{kj}\frac{\partial {g}_{2}}{\partial {Y}_{jp}}{X}_{pi}-{Y}_{ik}{X}_{kj}{Y}_{jp}\frac{\partial {g}_{2}}{\partial {X}_{pi}}\\ 	+\frac{\partial {g}_{2}}{\partial {Y}_{ik}}{X}_{kj}{X}_{jp}{Y}_{pi}+{Y}_{ik}\frac{\partial {g}_{2}}{\partial {X}_{kj}}{X}_{jp}{Y}_{pi}+{Y}_{ik}{X}_{kj}\frac{\partial {g}_{2}}{\partial {X}_{jp}}{Y}_{pi}+{Y}_{ik}{X}_{kj}{X}_{jp}\frac{\partial {g}_{2}}{\partial {Y}_{pi}}\\ 	+\frac{\partial {g}_{2}}{\partial {X}_{ik}}{Y}_{kj}{Y}_{jp}{X}_{pi}+{X}_{ik}\frac{\partial {g}_{2}}{\partial {Y}_{kj}}{Y}_{jp}{X}_{pi}+{X}_{ik}{Y}_{kj}\frac{\partial {g}_{2}}{\partial {Y}_{jp}}{X}_{pi}+{X}_{ik}{Y}_{kj}{Y}_{jp}\frac{\partial {g}_{2}}{\partial {X}_{pi}}\\ 	 - \frac{\partial {g}_{2}}{\partial {X}_{ik}}{Y}_{kj}{X}_{jp}{Y}_{pi}-{X}_{ik}\frac{\partial {g}_{2}}{\partial {Y}_{kj}}{X}_{jp}{Y}_{pi}-{X}_{ik}{Y}_{kj}\frac{\partial {g}_{2}}{\partial {X}_{jp}}{Y}_{pi}-{X}_{ik}{Y}_{kj}{X}_{jp}\frac{\partial {g}_{2}}{\partial {Y}_{pi}},$$

and we have written **E** = **X** + *i***Y** for real tensors **X** and **Y**. We then introduce a dynamics that minimizes our free energy above with respect to these constraints

16 $$\mu \frac{\partial {E}_{\alpha \beta }}{\partial t}=-\frac{\delta F}{\delta {E}_{\alpha \beta }^{*}}+{\delta }_{\alpha \beta }({\lambda }_{1a}+i{\lambda }_{1b})+{\lambda }_{2}\frac{\partial {g}_{2}}{\partial {E}_{\alpha \beta }^{*}}.$$

With respect to the main text,

17 $${{{\Lambda }}}_{\alpha \beta }={\delta }_{\alpha \beta }({\lambda }_{1a}+i{\lambda }_{1b})+{\lambda }_{2}\frac{\partial {g}_{2}}{\partial {E}_{\alpha \beta }^{*}}.$$

### Comparison to existing models

Substituting the eigenvalue *ψ* and vector **N** into Eq. (1) produces explicit forms for the free energy densities in terms of $\left|\psi \right|$, **∇***ϕ* and **N**. This allows us to compare to existing models. The bulk term (Eq. (2)) becomes

18 $${f}^{{{{{{{{\rm{bulk}}}}}}}}}=\frac{A\sigma }{2}{\left|\psi \right|}^{2}+\frac{C{\sigma }^{2}}{4}{\left|\psi \right|}^{4},$$

where $\sigma=\left(d-1\right)/d$, which demonstrates the consistency between this complex tensor theory approach and scalar-based bulk free energies^67^. Other possible real terms of the form $\sim {{{{{{{\rm{tr}}}}}}}}{({{{{{{{\bf{E}}}}}}}}+{{{{{{{{\bf{E}}}}}}}}}^{*})}^{\beta }$ are zero, while terms of the form $\sim [{{{{{{{\bf{E}}}}}}}}+{{{{{{{{\bf{E}}}}}}}}}^{*}]:[{{{{{{{\bf{E}}}}}}}}+{{{{{{{{\bf{E}}}}}}}}}^{*}]$ depend directly on *ϕ*, which is non-physical. Therefore, such terms cannot be included in Eq. (2). The compression term (Eq. (3)) becomes

19 $$\frac{{f}^{{{{{{{{\rm{el}}}}}}}}}}{{b}_{1}{\left|\psi \right|}^{2}}=\left(\frac{{{{{{{{\boldsymbol{\nabla }}}}}}}}\left|\psi \right|}{\left|\psi \right|}\right)\cdot \left(\frac{{{{{{{{\boldsymbol{\nabla }}}}}}}}\left|\psi \right|}{\left|\psi \right|}\right)+\left({{{{{{{\boldsymbol{\nabla }}}}}}}}\phi \right)\cdot \left({{{{{{{\boldsymbol{\nabla }}}}}}}}\phi \right)+\left({{{{{{{\boldsymbol{\nabla }}}}}}}}{{{{{{{\bf{N}}}}}}}}\right):\left({{{{{{{\boldsymbol{\nabla }}}}}}}}{{{{{{{\bf{N}}}}}}}}\right),$$

where we have make use of the identity *N*_*i*_∇_*j*_*N*_*i*_ = 0, which is due to the fact that **N** is a unit vector. Differentiating this again, we see that $\left({\nabla }_{k}{N}_{i}\right)\left({\nabla }_{j}{N}_{i}\right)+\left({N}_{i}{\nabla }_{k}\right)\left({\nabla }_{j}{N}_{i}\right)=0$, which we make use of below. Finally, the curvature term (Eq. (4)) is the most complicated, becoming

20 $$\frac{{f}^{{{{{{{{\rm{curv}}}}}}}}}}{{b}_{2}{\left|\psi \right|}^{2}}	=\sigma \left[{\left(\frac{{\nabla }^{2}\left|\psi \right|}{\left|\psi \right|}\right)}^{2}+{\left({\nabla }^{2}\phi \right)}^{2}+{\left({{{{{{{\boldsymbol{\nabla }}}}}}}}\phi \cdot {{{{{{{\boldsymbol{\nabla }}}}}}}}\phi \right)}^{2} \right.\\ 	 \; \; \; \left.+2\left\{\left({\nabla }^{2}\phi \right)\frac{{{{{{{{\boldsymbol{\nabla }}}}}}}}\left|\psi \right|}{\left|\psi \right|}-\left(\frac{{\nabla }^{2}\left|\psi \right|}{\left|\psi \right|}\right){{{{{{{\boldsymbol{\nabla }}}}}}}}\phi \right\}\cdot {{{{{{{\boldsymbol{\nabla }}}}}}}}\phi+4{\left\{\left(\frac{{{{{{{{\boldsymbol{\nabla }}}}}}}}\left|\psi \right|}{\left|\psi \right|}\right)\cdot {{{{{{{\boldsymbol{\nabla }}}}}}}}\phi \right\}}^{2}\right]\\ 	\quad+8\left[\left(\left\{\frac{{{{{{{{\boldsymbol{\nabla }}}}}}}}\left|\psi \right|}{\left|\psi \right|}\cdot {{{{{{{\boldsymbol{\nabla }}}}}}}}\right\}{{{{{{{\bf{N}}}}}}}}\right)\cdot \left(\left\{\frac{{{{{{{{\boldsymbol{\nabla }}}}}}}}\left|\psi \right|}{\left|\psi \right|}\cdot {{{{{{{\boldsymbol{\nabla }}}}}}}}\right\}{{{{{{{\bf{N}}}}}}}}\right) \right.\\ 	\quad \left.+\left(\left\{{{{{{{{\boldsymbol{\nabla }}}}}}}}\phi \cdot {{{{{{{\boldsymbol{\nabla }}}}}}}}\right\}{{{{{{{\bf{N}}}}}}}}\right)\cdot \left(\left\{{{{{{{{\boldsymbol{\nabla }}}}}}}}\phi \cdot {{{{{{{\boldsymbol{\nabla }}}}}}}}\right\}{{{{{{{\bf{N}}}}}}}}\right)+\left(\left\{\frac{{{{{{{{\boldsymbol{\nabla }}}}}}}}\left|\psi \right|}{\left|\psi \right|}\cdot {{{{{{{\boldsymbol{\nabla }}}}}}}}\right\}{{{{{{{\bf{N}}}}}}}}\right)\cdot \left({\nabla }^{2}{{{{{{{\bf{N}}}}}}}}\right)\right]\\ 	\quad+4\left({{{{{{{\bf{N}}}}}}}}\cdot {\nabla }^{2}{{{{{{{\bf{N}}}}}}}}\right)\left(\frac{{\nabla }^{2}\left|\psi \right|}{\left|\psi \right|}-{{{{{{{\boldsymbol{\nabla }}}}}}}}\phi \cdot {{{{{{{\boldsymbol{\nabla }}}}}}}}\phi \right) \\ 	\quad+2\left[\left({\nabla }^{2}{{{{{{{\bf{N}}}}}}}}\right)\cdot \left({\nabla }^{2}{{{{{{{\bf{N}}}}}}}}\right)+\left({\nabla }_{k}{{{{{{{\bf{N}}}}}}}}{\nabla }_{k}{{{{{{{\bf{N}}}}}}}}\right):\left({\nabla }_{\ell }{{{{{{{\bf{N}}}}}}}}{\nabla }_{\ell }{{{{{{{\bf{N}}}}}}}}\right)\right],$$

where we have stated the contraction on the gradients in the last term using Einstein notation for clarity but have used vector notation elsewhere. It is worth re-emphasizing that none of the contributions to the free energy density depend directly on the phase *ϕ*. In these forms, the **E**-tensor theory can be compared to existing models.

In the ground state equilibrium of flat, equally spaced, layers, the deformation free energies (Eq. (19) and Eq. (20)) are both zero. Only the bulk free energy *f* ^bulk^ (Eq. (18)) is non-zero and its form is consistent with scalar-based bulk free energies^36,37,67^.

In the limit of fixed *ψ* but variable **N**, only incompressible distortions are allowed. Taking *ψ* to be constant, the deformation free energies (Eq. (19) and Eq. (20)) become

21 $$\frac{{f}^{{{{{{{{\rm{el}}}}}}}}}}{{\left|\psi \right|}^{2}{b}_{1}} \, \approx \, \left({{{{{{{\boldsymbol{\nabla }}}}}}}}{{{{{{{\bf{N}}}}}}}}\right):\left({{{{{{{\boldsymbol{\nabla }}}}}}}}{{{{{{{\bf{N}}}}}}}}\right)$$

22 $$\frac{{f}^{{{{{{{{\rm{curv}}}}}}}}}}{2{\left|\psi \right|}^{2}{b}_{2}} \, \approx \, \left({\nabla }^{2}{{{{{{{\bf{N}}}}}}}}\right)\cdot \left({\nabla }^{2}{{{{{{{\bf{N}}}}}}}}\right)+\left({\nabla }_{k}{{{{{{{\bf{N}}}}}}}}{\nabla }_{k}{{{{{{{\bf{N}}}}}}}}\right):\left({\nabla }_{\ell }{{{{{{{\bf{N}}}}}}}}{\nabla }_{\ell }{{{{{{{\bf{N}}}}}}}}\right).$$

If we further only keep the lowest order term in the deformation free energy $\sim \left({{{{{{{\boldsymbol{\nabla }}}}}}}}{{{{{{{\bf{N}}}}}}}}\right):\left({{{{{{{\boldsymbol{\nabla }}}}}}}}{{{{{{{\bf{N}}}}}}}}\right)$, then this is precisely the nematic deformation free energy in the one-constant approximation^70^. We note that this specifically does not lead to the Oseen constraint that twist be prohibited, a prevalent simplifying assumption in models of smectic materials, because we have explicitly made a one-constant elastic approximation for simplicity. In nematic models near the vicinity of the nematic-smectic A phase transition, the ratios of elastic constants significantly differ from unity^96^. More intricate **E**-tensor theories that allow for differing elasticities will be able to make twist and bend of the layer normal (splaying of the layers themselves) come at a increased free energy cost.

We next presume that the layer normal is fixed globally along a constant axis and $\left|\psi \right|$ is constant, representing a smectic that is sufficiently deep within the lamellar phase. In this case, the deformation free energies (Eq. (19) and Eq. (20)) become

23 $$\frac{{f}^{{{{{{{{\rm{el}}}}}}}}}}{{\left|\psi \right|}^{2}{b}_{1}}=\left({{{{{{{\boldsymbol{\nabla }}}}}}}}\phi \right)\cdot \left({{{{{{{\boldsymbol{\nabla }}}}}}}}\phi \right)$$

24 $$\frac{{f}^{{{{{{{{\rm{curv}}}}}}}}}}{\sigma {\left|\psi \right|}^{2}{b}_{2}}={\left({\nabla }^{2}\phi \right)}^{2}+{\left({{{{{{{\boldsymbol{\nabla }}}}}}}}\phi \cdot {{{{{{{\boldsymbol{\nabla }}}}}}}}\phi \right)}^{2},$$

which together have the form ${c}_{0}+{B}_{1}{\left|\nabla \phi \right|}^{2}+{B}_{2}{\left|\nabla \phi \right|}^{4}+K{({\nabla }^{2}\phi )}^{2}$, where we have included an arbitrary constant, assigned the *f* ^curv^ differing elastic coefficients, and absorbed ${\left|\psi \right|}^{2}$ into each coefficient. Moreover, for simplifying choices of constants, this becomes $f=B{(1-{\left|\nabla \phi \right|}^{2})}^{2}+K{({\nabla }^{2}\phi )}^{2}$, which is an existing model free energy density for smectics^7,13,71,97^. We could also choose to write this in terms of a layer displacement *u* = *ϕ*/*q*_0_, making this free energy $f=B{q}_{0}^{2}{\left(\left|{{{{{{{\boldsymbol{\nabla }}}}}}}}u\right|-1\right)}^{2}+K{q}_{0}^{4}{({\nabla }^{2}u)}^{2}$. Models of this form are used to describe smectic systems^98^ and are reminiscent of the de Gennes–McMillan form^20,99^, although here we have made one-constant approximations for elasticity in the parallel and perpendicular directions. Furthermore, if the higher-order $\sim {\left({{{{{{{\boldsymbol{\nabla }}}}}}}}\phi \cdot {{{{{{{\boldsymbol{\nabla }}}}}}}}\phi \right)}^{2}$ term is neglected then Eqs. (23)-(24) reduces to the Brazovskii free energy, which is commonly employed to model for lamellar diblock copolymers^100–107^.

### Biaxiality

In the present study, we have exclusively focused on 2D systems for which uniaxiality is maintained due to the tracelessness constraint (requiring eigenvalues to be equal and opposite). However, a degree of biaxiality could be conceived along a secondary direction in 3D. In three dimensional nematic liquid crystals, allowing biaxiality increases the number of degrees of freedom in the order parameter **Q** from three in the uniaxial case to five. The two ancillary degrees of freedom represent the degree of biaxial alignment and the biaxial direction, constrained to be both a unit vector and orthogonal to the director. However, bulk biaxiality at equilibrium requires higher order terms in the expansion of the bulk free energy^64^. On the other hand, the higher order terms are not needed for biaxiality to naturally emerge in nematic defect core regions^108^. This is because the three eigenvalues can be distinct in regions where the ordering goes to zero^109^ and in strong confinements^110^. Similarly, biaxiality in **E** increases the degrees of freedom from four in the uniaxial case to seven. The three extra degrees of freedom represent another degree of ordering, biaxial layer displacement and the secondary direction, constrained to be a unit vector orthogonal to **N**. The biaxial case will not necessarily have a uniform complex phase across different components of the tensor and biaxiality at equilibrium would require higher order terms in the bulk free energy expansion (Eq. (2)).

### Defect identification

To identify defects, we measure winding numbers

25 $${m}_{\alpha }={(2\pi )}^{-1}\oint _{{{\Gamma }}}d\alpha$$

at each point over the smallest possible closed loop Γ, as a measure of topological charge. For disclinations, we measure the change in the layer normal azimuthal angle, *dα* = **N** ⋅ *d***l**. Values of *m*_**N**_ = ± 1/2 correspond to half-charge topological defects. For dislocations, *α* = *ϕ* measures the displacement of layers and gives the strength of the Burgers vector.

### Layer visualization

To visualize the layers, we use

26 $${{{{{{{\rm{Re}}}}}}}}\left[{{\Psi }}\right]=\left|\psi \right|\cos ({q}_{0}{{{{{{{\bf{N}}}}}}}}\cdot {{{{{{{\bf{r}}}}}}}}+\phi ).$$

Note however that this visualization technique does not respect the smectic symmetry; the focus is on providing an intuitive visual description of the layer structure. To calculate ${{{{{{{\rm{Re}}}}}}}}\left[{{\Psi }}\right]$, we use a Voronoi transformation of the plane into regions by defects and use the location of each defect as the origin for the dot product in Eq. (26). This numerical scheme gives good visualization of isolated defects in terms of layer structure (see Fig. 1c–e) but the transition from smooth $\left|\psi \right|$ and *ϕ* fields can introduce aberrations in defect-crowed regimes [see Fig. 3c]. This is due to the ambiguity of the layer displacement that is not possessed when working in terms of **E** alone. Visualizations based on ${{{{{{{\rm{Re}}}}}}}}\left[{{\Psi }}\right]$ must necessarily re-introduce the shortcomings that **E**-theory bypasses and so we generally avoid visualizations of ${{{{{{{\rm{Re}}}}}}}}\left[{{\Psi }}\right]$, except as a qualitative guide. Despite these disadvantages, we at times find it convenient to have an explicit, if rough, visualization of the layer configuration. The advantage of working with these fields can be seen in Fig. 3f, g and Supplementary Movies 2, 3. The resulting layer visualization is in Fig. 3c and Supplementary Movie 4.

## Supplementary information

Supplementary Information

Description of Additional Supplementary Files

Supplementary Movie 1

Supplementary Movie 2

Supplementary Movie 3

Supplementary Movie 4

Supplementary Movie 5

Supplementary Movie 6

## Data Availability

All data generated in this study are included in this published article (and its supplementary information files).

## Code availability

Codes are available upon request.
