# Supplementary material for: Complex-tensor theory of simple smectics
Source: Nat Commun. 2023 Feb 24;14:1048. doi: 10.1038/s41467-023-36506-z (PMC9958025; doi:10.1038/s41467-023-36506-z)
Supplement: Supplementary file 1 — Supplementary Information [file 41467_2023_36506_MOESM1_ESM.pdf]

Supplementary Material
Complex-tensor theory of simple smectics

Jack Paget,[1] Marco G. Mazza,[1,2] Andew J. Archer,[1] and Tyler N. Shendruk[3,*]

[1] *Interdisciplinary Centre for Mathematical Modelling and Department of Mathematical Sciences, Loughborough University, Loughborough, Leicestershire LE11 3TU, UK.*

[2] *Max Planck Institute for Dynamics and Self-Organization (MPIDS), Am Faßberg 17, D-37077 Göttingen, Germany.*

[3] *School of Physics and Astronomy, The University of Edinburgh, Peter Guthrie Tait Road, Edinburgh, EH9 3FD, UK.*

* t.shendruk@ed.ac.uk

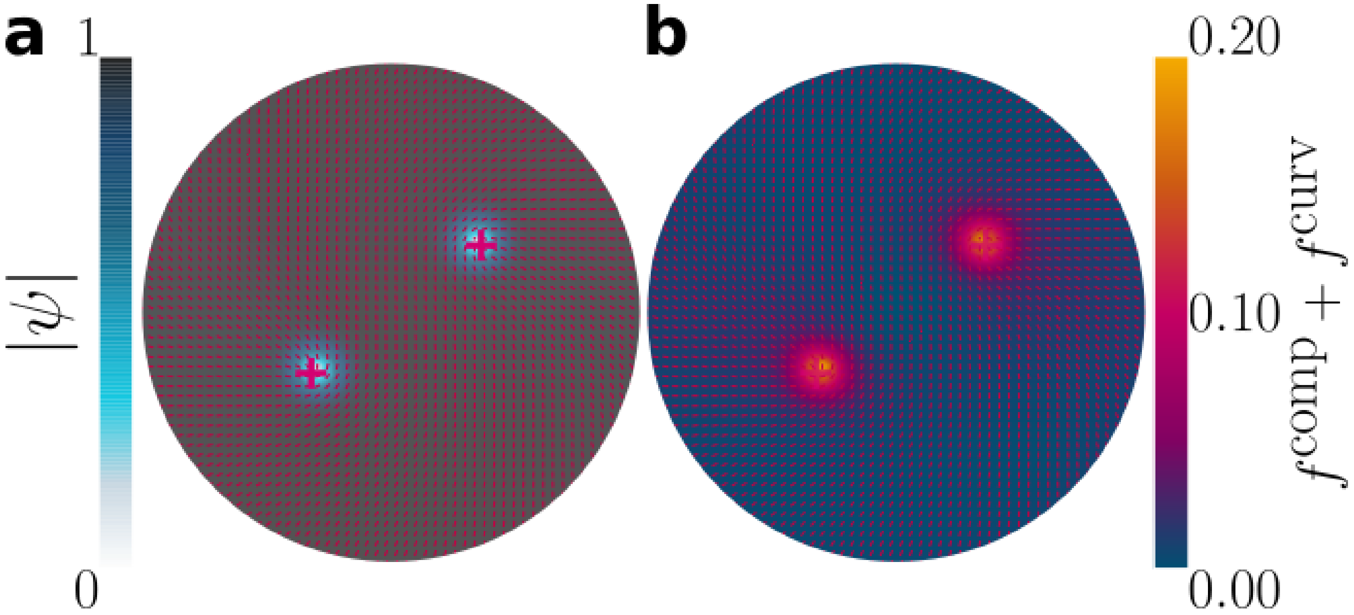

SUPPLEMENTARY FIG. 1. **The free energy density deviates from the bulk value only in the vicinity of the defects, *i.e.* no unphysical line is present.** Circular domain showing two confined +1/2 disclinations. For lamellae with $A = -1$ (lamellar phase), $C = 2$ and $\kappa^2 = 0.75$. Plus-half (+1/2) disclinations marked by red crosses. **(a)** Modulus $|\psi|$ showing melting around each defect. **(b)** Elastic free energy density normalized by $|A|$, showing peaks at the defects and no free energy line connecting them.

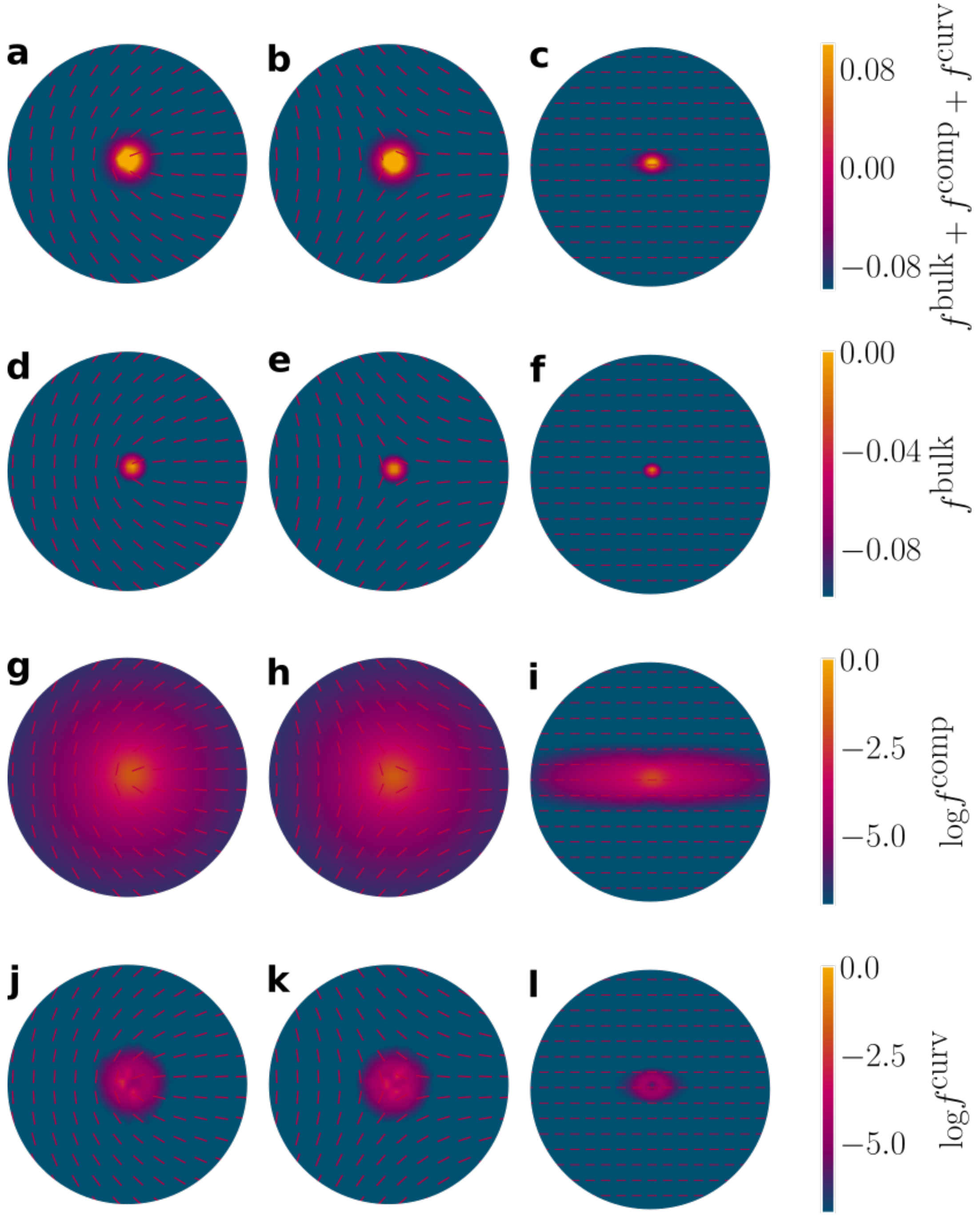

SUPPLEMENTARY FIG. 2. **Different terms of the free energy density for each defect type.** Normalized free energy density within circular domains with boundary conditions requiring single defects. The three columns depicted here match the columns in Fig. 1, for lamellae with $A = -1$ (lamellar phase), $C = 2$ and $\kappa^2 = 0.75$. Free energy densities normalized by $|A|$. **Columns** present three defect types: **(a,d,g,j)** Plus-half (+1/2) disclination defect; **(b,e,h,k)** Minus-half (−1/2) disclination; **(c,f,i,l)** Edge dislocation defect. **Rows** show free energy density contributions normalized by $|A|$ for each. **(a,b,c)** Total free energy density f for (a) plus-half disclination, (b) minus-half disclination, (c) edge dislocation. **(d,e,f)** Bulk $f^{\rm bulk}$ from Eq. (2) for (d) plus-half disclination, (e) minus-half disclination, (f) edge dislocation. **(g,h,i)** Compression $f^{\rm el}$ from Eq. (3) for (g) plus-half disclination, (h) minus-half disclination, (i) edge dislocation. **(j,k,l)** Curvature $f^{\rm curv}$ from Eq. (4) for (j) plus-half disclination, (k) minus-half disclination, (l) edge dislocation.

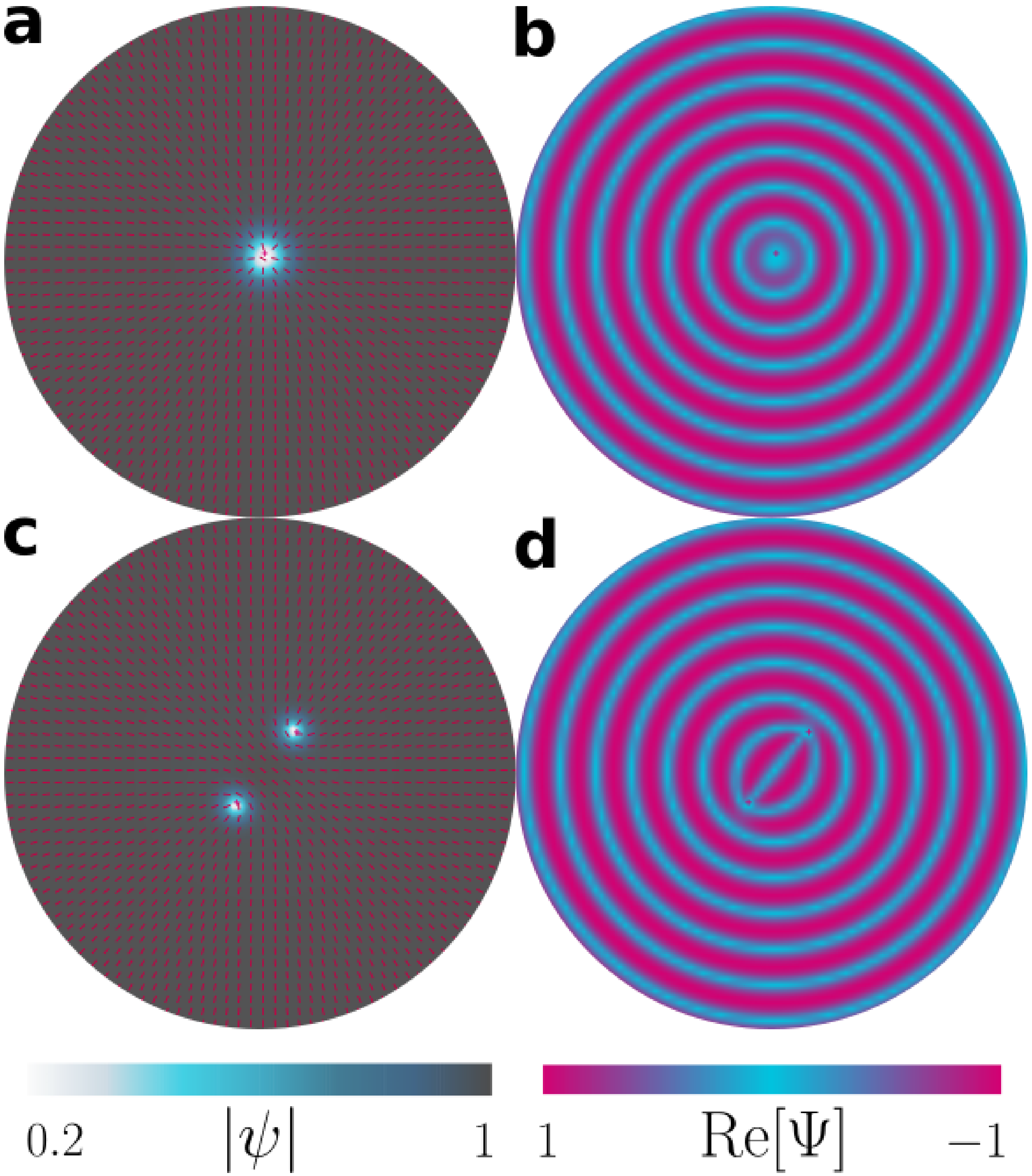

SUPPLEMENTARY FIG. 3. **Transition of +1 to arrested +1/2 pair in circular confinement.** A system in the lamellar phase ($A = -1$, $C = 2$ and $\kappa^2 = 0.6$) in a circular domain with homeotropic anchoring of the layer normal, and initialised as shown in Fig. 2c. This disordered region in the centre forms a +1 disclination ((**a**) and (**b**), $t = 2.5\mu$) before relaxing to an equilibrium pair of separated +1/2 disclinations ((**c**) and (**d**), $t = 50\mu$). Plots show the amplitude, $|\psi|$ ((**a**) and (**c**)) and $\mathrm{Re}[\Psi]$ ((**c**) and (**d**)).

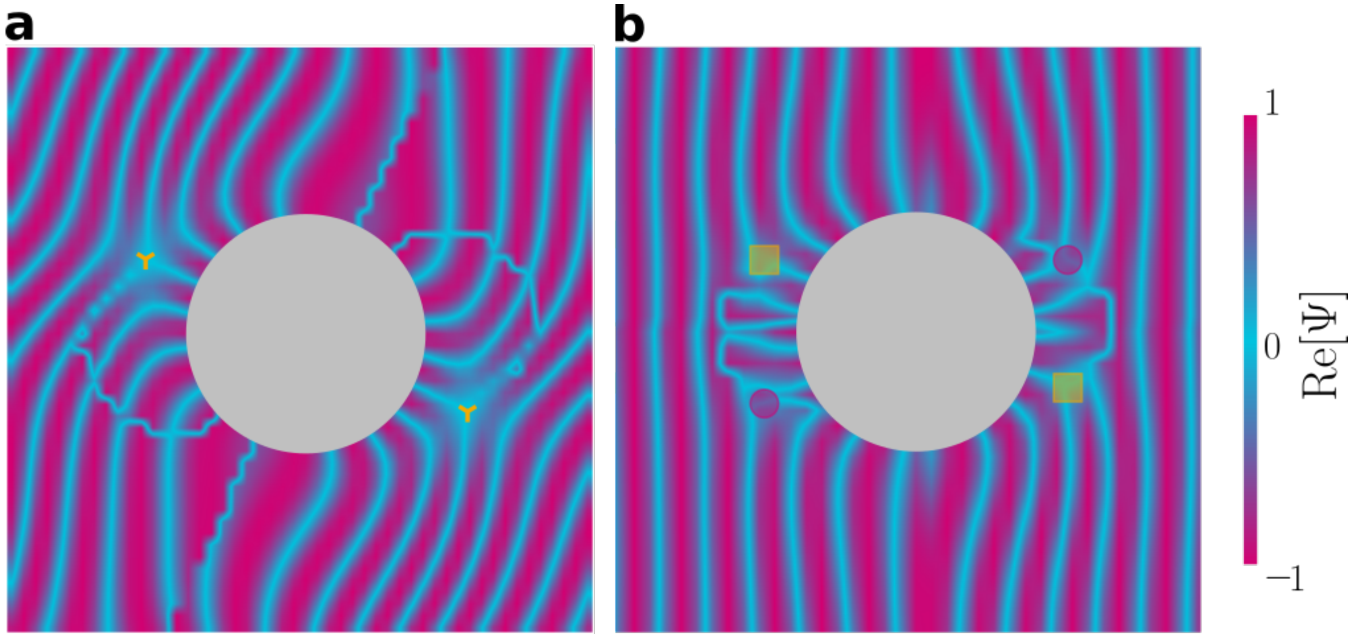

SUPPLEMENTARY FIG. 4. **Plots of Re [Ψ] for defects around a colloid.** These plots give a qualitative representation of the lamellar structure shown by ψ and $\mathbf{N}$ in Fig. 4c and Fig. 5. Since the $\mathbf{N} \to -\mathbf{N}$ symmetry is not respected by Re [Ψ], these post-processing visualizations introduce rendering aberrations that $\mathbf{E}$ does not possess. **(a)** Re [Ψ] to accompany Fig. 4c. **(b)** Re [Ψ] for Fig. 5.

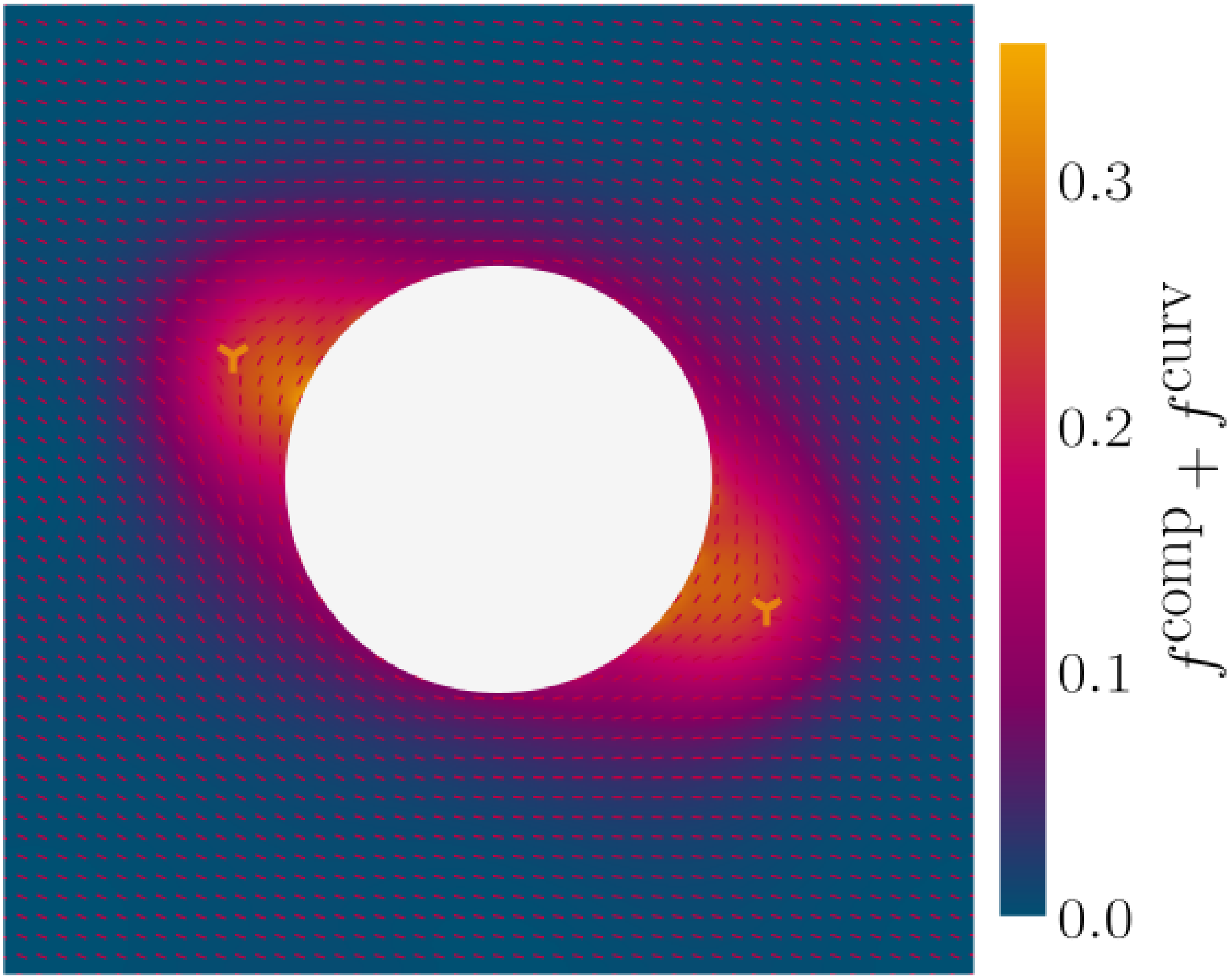

SUPPLEMENTARY FIG. 5. **Free energy density around a colloid with two satellite defects.** Normalized deformation free energy density (excludes f^{bulk}) shown for a colloidal inclusion in a smectic (same conditions as Fig. 4c and Supplementary Movie 6). Yellow trilaterals mark $-1/2$ disclinations and $\mathbf{N}$ is shown by the red vector field plot. For lamellae with $A = -1$ (lamellar phase), $C = 2$ and $\kappa^2 = 0.5$. Free energy densities normalized by $|A|$.
