# Supplementary material for: Complex-tensor theory of simple smectics
Source: Nat Commun. 2023 Feb 24;14:1048. doi: 10.1038/s41467-023-36506-z (PMC9958025; doi:10.1038/s41467-023-36506-z)
Supplement: Supplementary file 2 — Description of Additional Supplementary Files [file 41467_2023_36506_MOESM2_ESM.pdf]

Description of Additional Supplementary Files

File Name: Supplementary Movie 1
Description: Relaxation dynamics of lamellar order field $|\psi|$ for a $14\xi \times 14\xi$ system. The system is initialized in a disordered state with random layer normal, N, and order parameter, ψ. From this quenched state, the system finds the global free energy minimum. Time is in units of μ. A snapshot from this movie is shown in Fig. 3e-inset. Parameters are $A = -1$, $C = 2$, $\kappa^2 = 0.5$ with periodic boundary conditions. (legend) In all movies, red crosses denote +1/2 disclinations, trilateral mark −1/2 disclinations, and edge dislocations with winding number ±1 denoted by pink circles and yellow squares respectively.

File Name: Supplementary Movie 2
Description: Same as Movie 1 but for a larger system size of $42\xi \times 42\xi$ system. Pink crosses (yellow trilaterals) mark +1/2 (−1/2) disclinations. Edge dislocations with winding number ±1 denoted by pink circles and yellow squares. This system relaxes from a quenched disordered initial state to a frustrated local free energy minimum configuration, a glassy state with embedded defects. A snapshot from this movie is shown in Fig. 3g. Parameters are $A = -1$, $C = 2$, $\kappa^2 = 0.5$ with periodic boundary conditions.

File Name: Supplementary Movie 3
Description: Relaxation dynamics of the phase field ϕ for the same system as in Movie 2. Defects are marked in the same manner as Movie 2. A snapshot from this movie is shown in Fig. 3f.

File Name: Supplementary Movie 4
Description: Relaxation dynamics of the Re $[\Psi]$ field for the same system as in Movie 2. Defects are marked in the same manner as Movie 2. Snapshots from this movie are shown in Fig. 3a-c.

File Name: Supplementary Movie 5
Description: Relaxation dynamics of the deformation free energy contributions, $f_{el} + f_{curv}$ for the same system as in Movie 2. Defects are marked in the same manner as Movie 2.

File Name: Supplementary Movie 6
Description: Relaxation dynamics of lamellar order field $|\psi|$ for a $20\xi \times 20\xi$ system with an embedded circular inclusion. The steady state is shown in Fig. 4c. The inclusion radius is $R = 4\xi$ with strong anchoring of $\psi = e^{i\pi/2}$ and the layer normal **N** parallel to the surface. Time is in units of μ. Yellow trilaterals mark −1/2 disclinations and **N** is shown by the red vector field plot. Parameters are $A = -1$, $C = 2$, $\kappa^2 = 0.5$, $R = 4\xi$, with periodic boundary conditions.
